# Climate for Consumptives

**Published:** 1882-09

**Authors:** 


Climate for Consumptives.—It has been a fashion of many years' standing, to go to the South, when the lungs seemed to be affected; or to take long journeys by sea. Of late years, another "notion" seems to have taken hold of the public mind, to wit, that Minnesota, the great North-West, is best adapted toward recovering a man from consumption; and now, the stream of consumptive travelers is in that direction, instead of toward the sunny South, to Cuba, Madeira, and other localities. This change of sentiment originated in loose newspaper statements, that very few persons were noticed to have died of consumption in Minnesota. Similar statements have been made as to California, and for the very same reasons; both countries are comparatively new; few, other than the hardy, "settled" in them, and for obvious reasons the statistics on the subject must have been very imperfectly gathered. California is in a measure inaccessible, by reason of its distance. Havana and other warmer latitudes require more means than the multitude can command; hence, the great army moves toward the "North-West," with most discouraging results. The ablest resident physician at St. Paul, the chief town of Minnesota, says, that two thirds of the consumptives who reach that point, die there; and it is his frank and honorable habit to advise visitors to leave there as soon as possible. The air is indeed pure, and still and dry, having a uniform temperature in mid-winter; but whether from its great severity, or its rarefied character, or from its possessing some stranger ingredient, not yet detected, or whether from other causes, the fact remains the same, that two thirds of all who go to Minnesota for the removal of consumptive symptoms, perish there; and how many, soon after their return to their own homes, there are no means for ascertaining. But it is suggestive to note in the cases given in the preceding pages, that they were from all latitudes, from Canada to Cuba, leaving us to fall back on the great comprehensive fact, that the essential, the fundamental, the all-controlling agency in the arrest of any case of consumptive disease, and a return to reasonable health for any considerable time, is an active, courageous, and hopeful out-door life, in all weathers and in any latitude, with some rousing motive, other than regaining the health, beckoning them on, to do and to dare.

